# Restoring Tamoxifen Sensitivity in Breast Cancer: The Role of lncRNA MALAT1 and NanoCurcumin as Modulators of Drug Resistance

**DOI:** 10.1155/bmri/5824748

**Published:** 2025-09-02

**Authors:** Fatemeh Nasiri Kenari, Masoumeh Saberian, Matthew Abikenari, Safa Najafi, Majid Sadeghizadeh

**Affiliations:** ^1^ Department of Molecular Genetics, Faculty of Biological Science, Tarbiat Modares University, Tehran, Iran, modares.ac.ir; ^2^ Department of Hematology and Oncology, Valiasr Hospital, Tehran, Iran; ^3^ Department of Neurosurgery, Stanford University School of Medicine, Stanford, California, USA, stanford.edu; ^4^ Department of Oncology, Motamed Cancer Institute, Tehran, Iran

**Keywords:** breast cancer, lncRNA MALAT1, NanoCurcumin, plasma biomarker, tamoxifen resistance

## Abstract

**Background:** Tamoxifen resistance remains a major clinical challenge in estrogen receptor–positive (ER+) breast cancer, contributing to recurrence and poor prognosis. Long noncoding RNAs (lncRNAs), including MALAT1, UCA1, CYTOR, GAS5, and HOTAIR, have emerged as key regulators of endocrine resistance. Curcumin, a polyphenol with anticancer properties, modulates lncRNA expression, and its bioavailable formulation, NanoCurcumin, enhances therapeutic efficacy. This study evaluates the effects of NanoCurcumin in combination with tamoxifen on lncRNA expression and resistance mechanisms in ER+ breast cancer.

**Methods:** Plasma levels of the selected lncRNAs were assessed via qRT‐PCR in luminal breast cancer patients receiving tamoxifen alone or in combination with NanoCurcumin oral soft gels for 6 months. Bioinformatics analysis of MALAT1 expression was performed using the GEO database. In vitro, MALAT1 expression was evaluated in breast cancer (MCF7) and normal breast (MCF10) cell lines via qRT‐PCR. Tamoxifen‐resistant MCF7 cells were generated through prolonged treatment, and the effects of NanoCurcumin on MALAT1 expression were analyzed over 4 months.

**Results:** In clinical samples, NanoCurcumin significantly reduced MALAT1 expression (*p* = 0.02) and trended toward decreased UCA1, CYTOR, and HOTAIR while increasing GAS5 expression. Bioinformatics analysis confirmed MALAT1 upregulation in tamoxifen‐resistant cell lines. In vitro, MALAT1 was significantly elevated in MCF7 cells compared to MCF10 and increased over time with tamoxifen treatment alone. NanoCurcumin reversed this trend, sustaining low MALAT1 levels and mitigating resistance.

**Conclusion:** Our findings suggest that NanoCurcumin mitigates tamoxifen resistance by downregulating MALAT1, offering a novel epigenetic strategy to enhance endocrine therapy efficacy. Further studies should explore lncRNA‐targeted interventions to improve treatment outcomes in ER+ breast cancer.

## 1. Introduction

Breast cancer is a heterogeneous and multifactorial disease, resulting from the accumulation of genetic and epigenetic changes that transform normal cells into cancerous ones [1]. Approximately 70% of breast cancer patients have tumors with high levels of estrogen receptor alpha (ER*α*), which is targeted during hormone therapy. ER*α* promotes tumorigenesis and induces cell proliferation. Tamoxifen, a highly effective antiestrogen drug, is widely used in breast cancer patients with ER*α*‐positive status. It competitively blocks ER activation. Unfortunately, the effectiveness of hormone therapy is often compromised after long‐term treatment due to the development of hormonal resistance. Between 25% and 30% of patients are at risk of recurrence due to either intrinsic or acquired resistance to endocrine therapy [2, 3]. Resistance to endocrine therapies, such as tamoxifen, is a complex clinical challenge that frequently emerges during treatment, leading to recurrence, metastasis, and patient mortality. Dysregulation of long noncoding RNAs (lncRNAs) contributes to the development of tamoxifen resistance. lncRNAs play a vital role in regulating gene expression through direct interaction with mRNAs, noncoding RNAs (ncRNAs), and proteins [4, 5].

Curcumin, a natural polyphenol of *Curcuma longa* (turmeric), has drawn considerable attention owing to its excellent safety profile and broad spectrum of pharmacological activities that include anti‐inflammatory, antioxidant, antiproliferative, and proapoptotic activities [6–8]. Curcumin is shown to inhibit cell proliferation, migration, and invasion in several types of cancers, including breast, prostate, and brain cancers in in vitro and in vivo models [9–11]. Mechanistically, it suppresses the growth of tumors by regulating cell signaling pathways such as NF‐*κ*B, PI3K/Akt, and MAPK and inducing apoptosis through p53 activation and caspase cascade activation [8]. Curcumin is also reported to possess antiestrogenic activity through downregulating estrogen receptor expression and suppressing estrogen‐induced transcriptional activity [12]. Although its mechanism is distinct from that of the selective estrogen receptor modulators (SERMs) such as tamoxifen, both medications disrupt estrogen signaling and have demonstrated therapeutic usefulness in hormone‐responsive cancers [10–12]. This convergence of mechanisms underscores the broad usefulness of endocrine modulation as an effective principle in cancer therapy.

Research has shown that curcumin not only promotes cell death but also reinstates tamoxifen sensitivity in breast cancer cell lines resistant to antiestrogen therapy [10]. To enhance its solubility and bioavailability, this study utilized dendrosome nanoparticles. Dendrosomes, which are neutral, amphipathic, and biodegradable nanomaterials, were previously synthesized by our research team [13–17]. Additionally, recent findings suggest that curcumin exerts epigenetic modifications through ncRNAs in a variety of cancers [18]. ncRNAs are categorized into two groups based on their length: short ncRNAs and lncRNAs. lncRNAs are the largest class of endogenous ncRNAs, with lengths ranging from approximately 200 nucleotides to 100 kilobases. In the human genome, more than 50% of transcripts function as lncRNAs, which do not code for proteins but act directly as functional RNAs [5].

With the completion of the Human Genome Project, our understanding of the complexity and functional significance of lncRNAs has expanded considerably [19]. These transcripts, which do not code for proteins but play critical regulatory roles in gene expression, have been found to exhibit tissue‐specific and cancer‐specific expression patterns. Dysregulation of lncRNAs in tumor tissues compared to their nontumorous counterparts has been widely observed, highlighting their potential role in tumorigenesis, progression, and metastasis [20]. Given their stability in biological fluids, lncRNAs have garnered significant attention as promising biomarkers in liquid biopsy approaches. The analysis of circulating lncRNAs offers a minimally invasive yet highly sensitive method for detecting and monitoring complex diseases such as cancer, providing real‐time insights into disease dynamics and treatment response [21].

Several circulating lncRNAs, including *metastasis-associated lung adenocarcinoma transcript 1* (MALAT1), *urothelial carcinoma-associated 1* (UCA1), *HOX transcript antisense RNA* (HOTAIR), *cytoskeleton regulator RNA* (CYTOR), and *growth arrest-specific transcript 5* (GAS5), have demonstrated significant biomarker potential in breast cancer and other malignancies [22–26]. Among them, MALAT1 is a highly conserved lncRNA that plays a dual role in regulating gene expression at both transcriptional and posttranscriptional levels [27]. Notably, MALAT1 is frequently upregulated in breast cancer, where it exerts oncogenic effects by acting as a competing endogenous RNA (ceRNA). It directly sponges miR‐561‐3P, a known tumor suppressor, thereby promoting breast cancer cell proliferation, invasion, and metastasis [28].

Similarly, UCA1 has emerged as a key player in cancer progression and therapy resistance. Its role in conferring resistance to anticancer drugs has been well established across multiple cancer types [29]. In breast cancer, UCA1 induces tamoxifen resistance by activating critical oncogenic signaling pathways, including the mTOR and Wnt/*β*‐catenin pathways [30, 31]. Furthermore, UCA1 modulates drug resistance partly through the miR‐18a‐HIF1*α* feedback loop, further enhancing hypoxia‐induced tumor survival and metabolic adaptation [32]. Additionally, UCA1 regulates the EZH2/p21 axis and the PI3K/AKT signaling pathway, which are known to be pivotal in breast cancer progression and resistance to targeted therapies [33].

Another well‐characterized oncogenic lncRNA, HOTAIR, functions as a molecular scaffold for chromatin‐modifying complexes and is known to promote breast cancer progression by inhibiting tumor suppressor miRNAs while activating pro‐oncogenic miRNAs [34]. Mechanistically, HOTAIR acts as a ceRNA by sponging miR‐130a‐3p, leading to the derepression of *Suv39H1*‐mediated activation of the AKT/mTOR pathway, a critical regulator of cellular proliferation and survival [35].

Additionally, CYTOR has been implicated in breast cancer resistance to tamoxifen, where it enhances tumor cell survival by aberrantly activating the MAPK/ERK signaling cascade, a key pathway in tumor proliferation and drug resistance [4, 25]. Finally, GAS5, a tumor‐suppressive circulating lncRNA, plays an essential role in inhibiting cancer cell growth by acting as a decoy for glucocorticoid response elements and modulating apoptotic pathways. However, its expression is significantly suppressed in multiple malignancies, including breast cancer, suggesting that its downregulation may contribute to tumor progression and resistance to therapy [26, 36].

Collectively, these findings underscore the critical roles of circulating lncRNAs in breast cancer progression, therapy resistance, and biomarker potential. Their functional diversity and ability to modulate key oncogenic pathways highlight their promise as diagnostic, prognostic, and therapeutic targets in precision oncology. In addition to breast cancer, lncRNAs have also been shown to play important roles in central nervous system (CNS) tumors such as glioblastoma and meningioma [37–39]. In glioblastoma, lncRNAs like MALAT1 and HOTAIR contribute to tumor growth and resistance through similar immunosuppressive mechanisms within the tumor microenvironments seen in breast cancer [40–43]. Furthermore, curcumin has demonstrated antitumor effects in these brain tumors as well [44], suggesting that lncRNA‐targeted strategies using agents like curcumin may have broader applications beyond hormone‐sensitive cancers.

## 2. Materials and Methods

### 2.1. Clinical Study

#### 2.1.1. Study Design and Participants and Trial Treatment

This study was conducted on volunteer patients diagnosed with breast cancer who had undergone primary treatment, including surgery, chemotherapy, and radiotherapy, and subsequently initiated adjuvant endocrine therapy with tamoxifen. The study specifically focused on patients with luminal breast cancer subtypes, who have hormone receptor–positive (HR+) and human epidermal growth factor receptor 2 (HER2)–negative status, as these patients are typically treated with tamoxifen as part of standard hormone therapy. In contrast, HER2‐positive patients, who usually receive Herceptin (trastuzumab) in addition to tamoxifen, were excluded to maintain the study’s focus on endocrine therapy response in HER2‐negative cases.

In this trial, NanoCurcumin [13–17] was used as herbal medicine in the form of 35‐mg soft gelatin capsules with the brand name called Curcuden, which is registered by the Iran Food and Drug Administration (IRC: 4196698349576456) that is industrialized at Jaber Ebne Hayyan Pharmaceutical Company (Tehran, Iran).

Following volunteer patient selection, an initial blood sample was collected prior to the administration of tamoxifen. The study population was divided into two groups: the target group (*n* = 7, mean age = 49.6 years) received tamoxifen (10 mg twice daily) in combination with NanoCurcumin oral soft gels (35 mg twice daily), while the control group (*n* = 6, mean age = 43.8 years) received only tamoxifen at the same dosage. To minimize potential selection bias, patients from the target and control groups were recruited from two independent medical centers. A second blood sample was collected after 6 months of treatment to assess longitudinal changes in circulating biomarkers associated with tamoxifen resistance and response to NanoCurcumin supplementation.

#### 2.1.2. Ethics

The current study was approved by the Vice Chancellor of Research Affairs of Tarbiat Modares University (Code: IR. MODARES.REC.1401.001). Informed consent was signed by all study participants prior to conducting the trial. The study was conducted in accordance with all contemporary medical ethics practices, as well as policies outlined by the university and inherent to the field.

#### 2.1.3. Plasma Sample Collection and RNA Extraction

Peripheral blood samples were collected from both the target and control groups using BD Vacutainer Venous Blood Collection Tubes containing ethylenediaminetetraacetic acid (EDTA) as an anticoagulant. Immediately after collection, samples were stored at refrigerated temperatures and processed for plasma isolation. To achieve optimal plasma separation, samples were first subjected to centrifugation at 1900*g* for 10 min at 4°C, after which the plasma phase was carefully transferred to a fresh tube. To further remove residual cellular debris, a second centrifugation step was performed at 3000*g* for 15 min at 4°C (According to the miRNeasy Serum/Plasma Advanced Kit (QIAGEN) manufacturer’s standardized protocol). The clarified supernatant was then transferred into RNase‐ and DNase‐free microtubes and immediately stored at −80°C until further RNA extraction and analysis.

#### 2.1.4. RNA Extraction, cDNA Synthesis, and Real‐Time PCR

Total RNA was extracted from 200 *μ*L of plasma using the miRNeasy Serum/Plasma Advanced Kit (QIAGEN), following the manufacturer’s standardized protocol to ensure high RNA yield and purity. cDNA synthesis was performed using the RevertAid First Strand cDNA Synthesis Kit (Thermo Scientific) with both random hexamer and oligo‐dT primers to enhance the coverage of different RNA species.

For quantitative gene expression analysis, real‐time PCR (qPCR) was performed using the RealQ Plus 2x Master Mix Green, High Rox (Amplicon, Denmark). Reactions were carried out on an ABI Step One Sequence Detection System (Applied Biosystems, California, United States) under the following cycling conditions: 95°C for 20 s, 60°C for 20 s, and 72°C for 20 s, repeated for 40 cycles. Specific primers for the target genes were designed, validated, and ordered to Copenhagen, Denmark, with primer sequences and expected amplicon sizes detailed in Table 1. U6 small nuclear RNA was used as an internal control for normalization of gene expression levels [45].

**Table 1:** Primer sequences for real‐time PCR amplification of lncRNAs and U6 control. This table provides the primer sequences used for the real‐time PCR amplification of MALAT1, CYTOR, HOTAIR, UCA1, GAS5, and U6 (internal control). Total RNA was extracted from plasma samples using the miRNeasy Serum/Plasma Advanced Kit (QIAGEN), followed by cDNA synthesis with the RevertAid First Strand cDNA Synthesis Kit (Thermo Scientific). Real‐time PCR was performed using the ABI Step One System (Applied Biosystems, California, USA) with RealQ Plus 2x Master Mix Green, High Rox (Amplicon, Denmark). U6 was used as the endogenous control for normalizing lncRNA expression levels.

**Gene**	**Forward**	**Reverse**	**Product length**
U6	5 ^′^‐CGC TTC GGC AGC ACA TAT ACT A‐3 ^′^	5 ^′^‐ATG GAA CGC TTC ACG AAT TTG C‐3 ^′^	96 bp
MALAT1	5‐GATTTGAGCGGAAGAACGAATG‐3	5 ^′^‐TGCCATGTGCCTGGAATTA‐3 ^′^	96 bp
UCA1	5 ^′^‐CCT ATC TCC CTT CAC TGA CTC T‐3 ^′^	5 ^′^‐GTC CGT ATA GAA GAC ACC CAA TC‐3 ^′^	108 bp
HOTAIR	5 ^′^‐GCACTCACAGACAGAGGTTTAT‐3 ^′^	5 ^′^‐TGTACTCCCGTTCCCTAGATT‐3 ^′^	93 bp
GAS5	5 ^′^‐GAGCAAGCCTAACTCAAGCC‐3 ^′^	5 ^′^‐TCAAGCCGACTCTCCATACC‐3 ^′^	196 bp
CYTOR	5 ^′^‐ACCGAAAATCACGACTCAGCCC‐3 ^′^	5 ^′^‐AATGGGAAACCGACCAGACCAG‐3 ^′^	187 bp

By implementing stringent sample processing and qPCR methodologies, this study was aimed at assessing the differential expression of circulating lncRNAs and their potential role in modulating response to tamoxifen therapy, particularly in the presence of NanoCurcumin supplementation.

### 2.2. Bioinformatics Analysis

Expression data for lncRNA MALAT1 in tamoxifen‐sensitive and tamoxifen‐resistant breast cancer cells across four cell lines were extracted from the GEO database (https://www.ncbi.nlm.nih.gov/geo/) and using DESeq2.

### 2.3. In Vitro Studies

#### 2.3.1. Cell Culture

The MCF7 and MCF10 breast cancer cell line was obtained from the Pasteur Institute Cell Bank in Tehran. Cells were cultured in DMEM medium containing L‐glutamine (Zist Kala) and 10% fetal bovine serum (FBS) (Gibco), supplemented with 1% penicillin‐streptomycin (Zist Kala). The cells were incubated in a 5% CO_2_ atmosphere at 37°C. Once the cells reached 80% confluence, they were passaged.

#### 2.3.2. Treatment of MCF7 Cells With Tamoxifen and NanoCurcumin

To establish a tamoxifen‐resistant model, MCF7 cells were long‐term exposed to gradually increasing concentrations of tamoxifen (over 4 months) following our laboratory’s established protocol [46]. MALAT1 expression was monitored as a potential resistance‐associated marker [27, 28, 41, 47–53]. While IC_50_ or cell survival assays were not performed, transcriptomic profiling of MALAT1 served as a molecular readout of resistance acquisition.

To evaluate the combined effects of tamoxifen and NanoCurcumin, cells were cotreated with 10‐*μ*M NanoCurcumin and tamoxifen using the same escalation protocol. The NanoCurcumin concentration was selected based on our previous finding and other studies demonstrating its biological activity in inducing apoptosis and suppressing cancer cell proliferation [13, 16, 54, 55]. A formal dose–response analysis was not performed, as the objective was to assess coregulatory effects at a well‐tolerated and therapeutically relevant dose supported by literature.

Cells were cultured continuously under selective pressure and monitored monthly for changes in MALAT1 lncRNA expression profiles.

#### 2.3.3. RNA Extraction, cDNA Synthesis, and Real‐Time PCR

Total RNA was extracted using NORGEN total RNA purification kit according to the manufacturer’s instructions. RNA quality and quantity were assessed by gel electrophoresis and NanoDrop, respectively. RNA was treated with RNase‐free DNase 1 (Fermentase). cDNA synthesis and real‐time PCR were subsequently performed.

## 3. Results

### 3.1. Clinical Study Results

**Figure 1:**
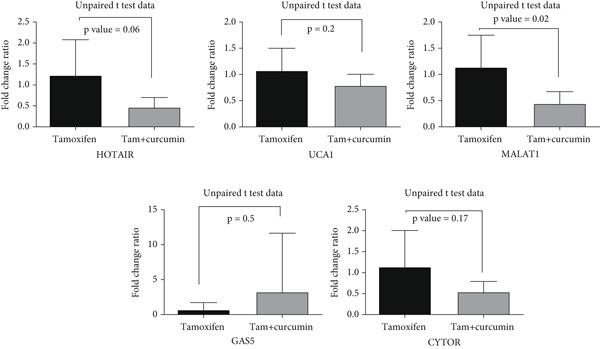
NanoCurcumin enhances tamoxifen response by modulating expression of lncRNAs associated with drug resistance in breast cancer cells. As shown in the graph, the combination of NanoCurcumin and tamoxifen resulted in a relative reduction in MALAT1, UCA1, HOTAIR, and CYTOR expression and a relative induction in GAS5 expression, which suggests a decrease in tamoxifen resistance. The bar graph presents the fold change ratio of MALAT1, UCA1, HOTAIR, CYTOR, and GAS5 expression in tamoxifen‐treated and tamoxifen + NanoCurcumin–treated groups. The results, analyzed using an unpaired *t*‐test, reveal a statistically significant difference in MALAT1 expression between the two groups (*p* = 0.02). In the UCA1, HOTAIR, and CYTOR expression, despite the lack of significance, there is a trend toward reduced expression, and in the GAS5 expression, there is a trend toward induced expression in the NanoCurcumin + tamoxifen group, potentially indicating a role for NanoCurcumin in modulating resistance mechanisms.

MALAT1, UCA1, CYTOR, HOTAIR, and GAS5 expressions were compared between breast cancer patients treated with tamoxifen alone (control) and those treated with tamoxifen plus NanoCurcumin (target group). Fold change in MALAT1, UCA1, CYTOR, HOTAIR, and GAS5 expression from Month 6 relative to Month 1 was calculated for each patient in both groups. The results indicated that the addition of NanoCurcumin to tamoxifen therapy led to a significant decrease in MALAT1 expression in the target group compared to the control group; expression changes in other lncRNAs were not significant, although they were in the direction we expected (Figure 1).

### 3.2. Bioinformatics Analysis

In this study, RNA sequencing and bioinformatics analysis were employed to assess the differential expression of lncRNAs in tamoxifen‐resistant versus tamoxifen‐sensitive cell lines. Data were obtained from the GEO database (https://ncbi.nlm.nih.gov/gene), specifically GSE111151 and GSE67916. lncMALAT1 was overexpressed in tamoxifen‐resistant cells across all cell lines (Table 2). This analysis confirms that lncMALAT1 is consistently upregulated in tamoxifen‐resistant MCF7, ZR75, BT474, and T47D cell lines, though statistical significance varies across datasets. Further validation is required to assess its functional role in resistance mechanisms.

**Table 2:** Differential expression of lncMALAT1 in tamoxifen‐resistant versus tamoxifen‐sensitive cell lines. This table presents the differential expression analysis of lncMALAT1 in tamoxifen‐resistant versus tamoxifen‐sensitive breast cancer cell lines. RNA sequencing data were retrieved from the GEO database (GSE111151 and GSE67916). The log2 fold change (Log2FC) values indicate the level of upregulation of lncMALAT1 in resistant cells; *p* values represent the statistical significance of differential expression.

**GSE excision number**	**Cell line**	**Status**	**Log**_**2**_ **(fold change)**	**p** **value**
111151	MCF7	Up	0.587327	0.375263
67916	MCF7	Up	0.5924560	1.4051e−04
111151	ZR75	Up	0.087598	0.749338
111151	BT474	Up	0.031072	0.920352
111151	T47D	Up	0.97432	0.089716

### 3.3. In Vitro Studies

#### 3.3.1. Comparison of Breast Cancer and Normal Breast Cell Lines

**Figure 2:**
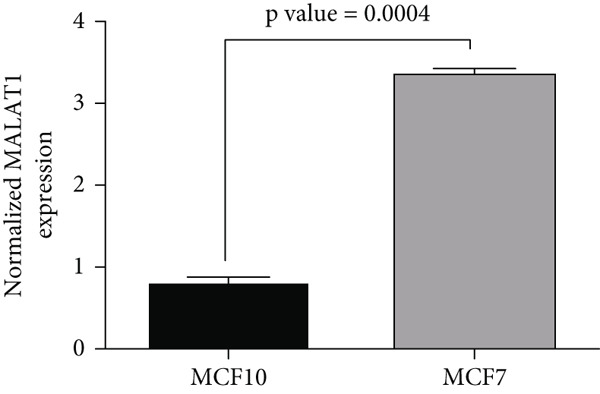
MALAT1 expression is significantly elevated in tamoxifen‐sensitive MCF7 breast cancer cells compared to nontumorigenic MCF‐10 cells. Quantitative real‐time PCR analysis revealed significantly higher expression of the long noncoding RNA MALAT1 in MCF7 estrogen receptor–positive breast cancer cells relative to nontumorigenic MCF‐10 mammary epithelial cells. Expression was normalized to U6 and is presented as mean ± SEM from three independent experiments. Statistical analysis was performed using unpaired two‐tailed Student’s *t*‐test, demonstrating a significant upregulation of MALAT1 in MCF7 cells (*p* = 0.0004). These findings support the oncogenic role of MALAT1 in hormone‐responsive breast cancer models.

We compared MALAT1 expression between the MCF7 breast cancer cell line and the normal breast cell line MCF10 using qRT‐PCR. MALAT1 expression was significantly higher in MCF7 compared to MCF10 (Figure 2).

#### 3.3.2. Changes in MALAT1 Expression After Tamoxifen and NanoCurcumin Treatment

**Figure 3:**
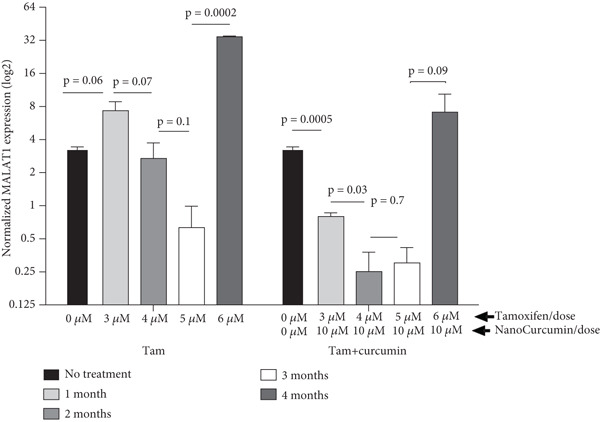
Time‐ and dose‐dependent effects of tamoxifen and NanoCurcumin on MALAT1 expression dynamics and potential drug resistance development. Normalized MALAT1 expression levels (log_2_) were quantified by qRT‐PCR in MCF7 cells across 4 months of treatment with either tamoxifen alone (with escalating doses over time) and tamoxifen combined with NanoCurcumin (fixed‐dose NanoCurcumin alongside escalating tamoxifen) monthly. In this study, we first performed a two‐way ANOVA to assess the effects of duration/dose of treatment and type of treatment on MALAT1 expression. The analysis revealed statistically significant effects for all main factors (*p* = 0.0001 for all). Following the significant result, we conducted unpaired *t*‐tests to analyze within‐group differences in gene expression. Untreated cells served as the initial baseline, and subsequent months were compared to their immediate previous time points. Data represent mean ± SEM of three biological replicates. MALAT1 expression decreased following tamoxifen treatment but increased after 4 months, potentially indicating the development of tamoxifen resistance. The persistent suppression of MALAT1 in the combination group across all time points and escalating tamoxifen doses suggests that NanoCurcumin prevents the accumulation of MALAT1, a lncRNA associated with tamoxifen resistance, offering mechanistic insight into its therapeutic synergy.

**Figure 4:**
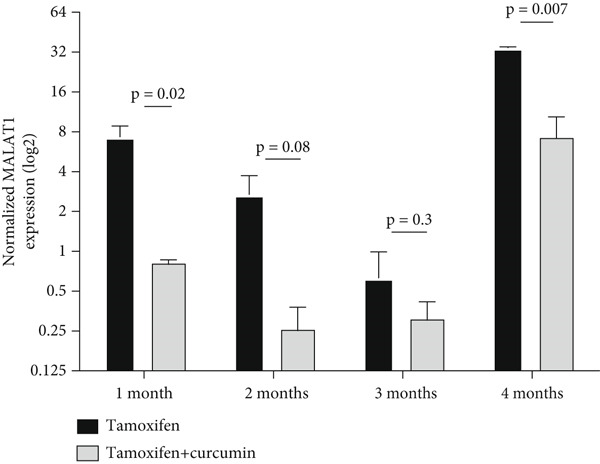
NanoCurcumin attenuates MALAT1 expression in tamoxifen‐treated MCF7 cells. This figure presents a time‐course comparison of MALAT1 expression (log_2_ normalized values) in MCF7 breast cancer cells treated with tamoxifen alone versus tamoxifen in combination with NanoCurcumin. Bar graphs show expression at 1, 2, 3, and 4 months of treatment. Black bars represent tamoxifen‐only groups, while gray checkered bars represent combined tamoxifen + NanoCurcumin treatment. At 1 month, MALAT1 was significantly reduced in the combination group compared to tamoxifen alone (*p* = 0.02). This suppressive trend persisted across 2, 3, and 4 months, with the most robust difference observed at 4 months (*p* = 0.007). While the 2‐month (*p* = 0.08) and 3‐month (*p* = 0.3) comparisons did not reach significance, they demonstrate a consistent downward trajectory in MALAT1 expression with NanoCurcumin. Statistical significance was assessed using unpaired two‐tailed Student’s ‐tests between treatment arms at each time point. Data represent the of three biological replicates. Together with Figure 3, this result provides orthogonal evidence that NanoCurcumin mitigates the MALAT1 expression, highlighting its potential to modulate long noncoding RNA–mediated resistance mechanisms in hormone receptor–positive breast cancer.

MCF7 cells were treated with tamoxifen alone and tamoxifen plus NanoCurcumin for 4 months. MALAT1 expression was evaluated monthly between the two groups. Data analysis using GraphPad software and statistical methods such as two‐way ANOVA and *t*‐tests revealed significant changes in MALAT1 expression over time. Initially, MALAT1 expression decreased in both groups, followed by an increase in expression after the fourth month (Figure 3). When comparing the two groups each month, MALAT1 expression decreased in the tamoxifen + curcumin group (Figure 4).

## 4. Discussion

### 4.1. The Challenge of Tamoxifen Resistance in Breast Cancer

Breast cancer remains a significant cause of cancer‐related mortality, with estrogen receptor–positive (ER+) subtypes comprising the majority of cases. Tamoxifen has been the mainstay of endocrine therapy for ER+ breast cancer; however, its long‐term efficacy is often compromised by the emergence of resistance, which occurs in 20%–30% of patients [1, 2]. This resistance not only undermines treatment outcomes but also contributes to disease recurrence and progression, necessitating the identification of novel therapeutic strategies to counteract this challenge. In recent years, accumulating evidence has highlighted the critical role of lncRNAs in breast cancer progression and therapeutic resistance [5, 20]. Among the numerous lncRNAs implicated in drug resistance, MALAT1, UCA1, CYTOR, and HOTAIR have emerged as crucial oncogenes, and GAS5 is a well‐known lncRNA that acts as a tumor suppressor in breast cancer [4, 20, 32, 33, 47, 48, 56].

The dysregulation of MALAT1 has been linked to poor prognosis in breast cancer, where its upregulation activates oncogenic pathways such as PI3K/AKT, Wnt/*β*‐catenin, and STAT3, leading to increased proliferation, survival, and therapeutic evasion [47, 49–51]. Our study provides compelling evidence that NanoCurcumin, a bioavailable formulation of curcumin, exerts a modulatory effect on MALAT1 expression and potentially reverses tamoxifen resistance, offering new mechanistic insights into how epigenetic regulation can reshape endocrine therapy response.

### 4.2. NanoCurcumin Suppresses MALAT1 and Modulates Other lncRNAs

Our clinical findings demonstrate that the combination of NanoCurcumin with tamoxifen results in a significant reduction in MALAT1 expression compared to tamoxifen alone, with a statistically significant *p* value of 0.02. This suggests that NanoCurcumin may mitigate the upregulation of MALAT1, which has been widely observed in endocrine‐resistant breast cancer [46, 47]. While reductions in other oncogenic lncRNAs, including UCA1, CYTOR, and HOTAIR, did not reach statistical significance, their downward trends, coupled with the upward trend of the tumor suppressor lncRNA GAS5, further reinforce the hypothesis that NanoCurcumin exerts a global effect on the lncRNA regulatory landscape [14]. These findings are consistent with previous studies demonstrating that MALAT1 is overexpressed in tamoxifen‐resistant breast cancer and is associated with poor response to therapy [49, 50]. Our bioinformatics analysis, leveraging publicly available gene expression datasets from the GEO database, further corroborates this notion, revealing that MALAT1 expression is consistently elevated in tamoxifen‐resistant breast cancer cell lines compared to their sensitive counterparts.

The in vitro experiments provide further mechanistic validation, illustrating that MALAT1 expression is markedly higher in MCF7 cells compared to normal breast epithelial MCF10 cells, suggesting a cancer‐specific role for MALAT1 in driving oncogenic processes. Importantly, our study elucidates the temporal dynamics of MALAT1 expression during prolonged tamoxifen treatment, demonstrating that while tamoxifen initially suppresses MALAT1 expression, its levels begin to rise again after 4 months, potentially marking a key transition toward resistance. The addition of NanoCurcumin effectively counteracted this resurgence, sustaining low MALAT1 levels throughout the treatment course. This finding is particularly relevant, as it suggests that MALAT1 upregulation may serve as an early biomarker of impending resistance, and its suppression via NanoCurcumin could offer a novel intervention strategy. Statistical analyses using two‐way ANOVA and unpaired *t*‐tests confirm that NanoCurcumin exerts a significant effect in reducing MALAT1 expression over time, reinforcing the potential of lncRNA‐targeted therapies as viable adjuncts to conventional endocrine treatments.

### 4.3. Mechanistic Insights: How NanoCurcumin Regulates MALAT1

The exact molecular mechanisms through which NanoCurcumin regulates MALAT1 expression remain an area of active investigation, though multiple lines of evidence suggest plausible pathways. MALAT1 is known to function as a ceRNA, sequestering tumor‐suppressive microRNAs such as miR‐561‐3P, miR‐125b, and miR‐218, thereby promoting oncogenic gene expression [20, 27, 28]. Given that curcumin has been shown to upregulate tumor‐suppressive microRNAs, it is conceivable that NanoCurcumin modulates MALAT1 expression via a microRNA‐mediated mechanism, effectively disrupting its oncogenic ceRNA function [10, 18]. Additionally, curcumin has been reported to inhibit key signaling cascades, including Wnt/*β*‐catenin and PI3K/AKT, both of which are known to be activated by MALAT1 in breast cancer [47, 51]. The suppression of these pathways by NanoCurcumin may lead to a downstream reduction in MALAT1 transcription, thereby curtailing its oncogenic effects. Furthermore, emerging evidence indicates that curcumin exerts epigenetic modifications, including alterations in DNA methylation and histone acetylation, which could further contribute to its inhibitory effect on MALAT1 expression [52, 53, 57]. These multifaceted mechanisms suggest that NanoCurcumin operates at multiple regulatory levels, reinforcing its therapeutic potential in overcoming endocrine resistance.

### 4.4. Clinical and Translational Implications of Our Findings

The translational implications of these findings are profound. As resistance to tamoxifen remains a major clinical hurdle, the integration of lncRNA‐modulating agents such as NanoCurcumin into therapeutic regimens may enhance treatment efficacy and prevent the emergence of resistance. The increasing availability of liquid biopsy technologies enables real‐time monitoring of circulating lncRNAs, presenting an opportunity for early detection of resistance markers such as MALAT1 and allowing for timely therapeutic interventions [21, 22]. Future studies should explore the integration of lncRNA‐based diagnostics with NanoCurcumin therapy to develop personalized treatment strategies tailored to individual resistance profiles. Additionally, expanding this research to include larger patient cohorts and long‐term follow‐up will be critical in validating the clinical utility of NanoCurcumin in endocrine therapy.

Our study highlights the promising role of NanoCurcumin in resensitizing tamoxifen‐resistant breast cancer by modulating epigenetic regulators such as MALAT1 [47, 52]. These findings align with previous reports demonstrating the therapeutic potential of curcumin in reversing chemoresistance, further supporting its incorporation into combination therapy approaches [10, 18]. The ability of NanoCurcumin to regulate multiple oncogenic pathways and epigenetic modifications suggests that its impact extends beyond MALAT1 alone, warranting further investigation into its broader effects on the ncRNA network.

Furthermore, emerging parallels between hormone‐driven tumors such as breast cancer and meningioma highlight a shared oncogenic reliance on PI3K/AKT signaling, lncRNA regulation, and hormonal receptor crosstalk [58–60]. A recent review on PIK3CA‐mutated meningiomas underscores that a subset of these tumors, particularly those with non‐NF2 mutations, exhibit estrogen and progesterone receptor positivity and respond aberrantly to hormonal exposure via PIK3CA pathway activation [59]. Similarly, in hormone‐sensitive breast cancers, PIK3CA mutations and PI3K/AKT pathway upregulation co‐occur with dysregulated lncRNAs like MALAT1 [58, 60], suggesting a conserved oncogenic axis. The ability of curcumin to modulate this axis across tumor types further supports its potential as a multicontextual therapeutic, targeting both hormonal signaling and downstream lncRNA effectors. Curcumin has also exhibited antitumoral properties in glioma models, including immunomodulatory and lncRNA‐modulatory effects, making it a plausible alternative for repurposing NanoCurcumin‐based therapies to enhance immunotherapy approaches such as CAR T‐cell therapy and immunometabolic changes [37, 40–42]. These results broaden the therapeutic horizon of lncRNA‐targeting therapies beyond breast cancer and position NanoCurcumin as a prime candidate to overcome resistance and restore immune competence in a broad spectrum of tumor settings.

### 4.5. Study Limitations and Considerations for Translation

Despite the promising findings presented, several limitations should be noted to contextualize the scope and generalizability of our results. First, the clinical sample was small (*n* = 13) and from a single‐region population, which limited statistical power and the ability to stratify responses by molecular subtypes or demographic subgroups. Second, although our study was aimed exclusively at HER2‐negative, HR+ breast cancer to maintain cohort homogeneity, this by definition restricts its translational relevance to other subtypes, that is, triple‐negative or HER2‐positive breast cancer. Third, although the NanoCurcumin dose used in vitro (10 *μ*M) was selected from prior studies and shown here to have a robust biological effect [13, 16, 54, 55], a dose–response curve and toxicity study were not performed formally. These analyses would provide more granular therapeutic window knowledge, especially in combination regimens. Finally, the clinical component of the study, though longitudinal, did not incorporate long‐term endpoints such as recurrence‐free or progression‐free survival, which are critical to the determination of durable therapeutic benefit. Future research must seek to validate these findings in larger multicenter cohorts with longer follow‐up, as well as include functional assays and mechanistic dissection to identify definitive causal pathways.

## 5. Conclusion: A Step Toward Personalized Cancer Therapies

In conclusion, our study underscores the critical role of MALAT1 in tamoxifen resistance and provides strong evidence that NanoCurcumin can serve as an effective modulator of MALAT1 expression, potentially reversing or preventing endocrine therapy resistance in ER+ breast cancer. The findings from our clinical, bioinformatics, and in vitro studies collectively highlight a compelling rationale for integrating NanoCurcumin into therapeutic strategies targeting lncRNAs. As research advances, the development of lncRNA‐directed therapies, combined with molecular diagnostics, may usher in a new era of precision medicine aimed at overcoming drug resistance and improving outcomes for breast cancer patients.

## Conflicts of Interest

The authors declare no conflicts of interest.

## Funding

The study is funded by the Vice Chancellor for Research and Technology of Tarbiat Modares University and the National Cancer Monitoring Charitable Foundation.

## Data Availability

The datasets used and analyzed during the current study are available from the corresponding author upon reasonable request.
